# Supplementary material for: Inhibition of jasmonate-mediated plant defences by the fungal metabolite higginsianin B
Source: J Exp Bot. 2020 Feb 1;71(10):2910–21. doi: 10.1093/jxb/eraa061 (PMC7260715; doi:10.1093/jxb/eraa061)
Supplement: eraa061_suppl_Supplementary_Figures_S1-S3 [file eraa061_suppl_supplementary_figures_s1-s3.pdf]

Inhibition of jasmonate-mediated plant defences by the fungal metabolite higginsianin B

Jean-Félix Dallery [1,4], Marlene Zimmer [2], Vivek Halder [3,§], Mohamed Suliman [3,†], Sandrine Pigné [1], Géraldine Le Goff [4], Despoina D. Gianniou [5], Ioannis P. Trougakos [5], Jamal Ouazzani [4], Debora Gasperini [2], Richard J. O'Connell [1]

[1] Université Paris-Saclay, INRAE, AgroParisTech, UMR BIOGER, 78850, Thiverval-Grignon, France

[2] Department of Molecular Signal Processing, Leibniz Institute of Plant Biochemistry, Halle (Saale), Germany

[3] Chemical Biology Laboratory, Max Planck Institute for Plant Breeding Research, Cologne, Germany

[4] Centre National de la Recherche Scientifique, Institut de Chimie des Substances Naturelles ICSN, 91198, Gif-sur-Yvette, France

[5] Department of Cell Biology and Biophysics, Faculty of Biology, National and Kapodistrian University of Athens, Greece

§ Current address: Rijk Zwaan, De Lier, 2678 ZG, Netherlands

†Current address: Desert Research Center, Cairo, Egypt

Corresponding authors: Richard J. O'Connell, richard.oconnell@inrae.fr; Debora Gasperini, debora.gasperini@ipb-halle.de

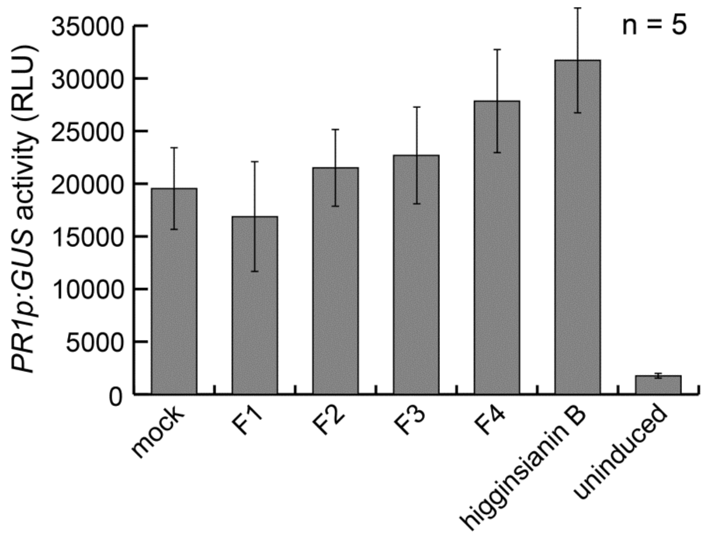

Supplementary Figure S1 – Screening assay for modulation of salicylic acid signalling pathway. *Arabidopsis* seedlings expressing GUS reporter under the control of *PR1* promoter, a marker of SA-mediated plant defences, were pre-treated with fractions or pure compound higginsianin B for 1h followed by SA treatment (200 µM) for 24 h. Bars represent means *PR1p:GUS* activity of 5 independent seedlings, ± SD from one representative experiment performed twice. None of the tested fractions or compound were significantly different from the mock control (adjusted *P*-value = 0.25, Kruskal-Wallis with Conover-Iman test).

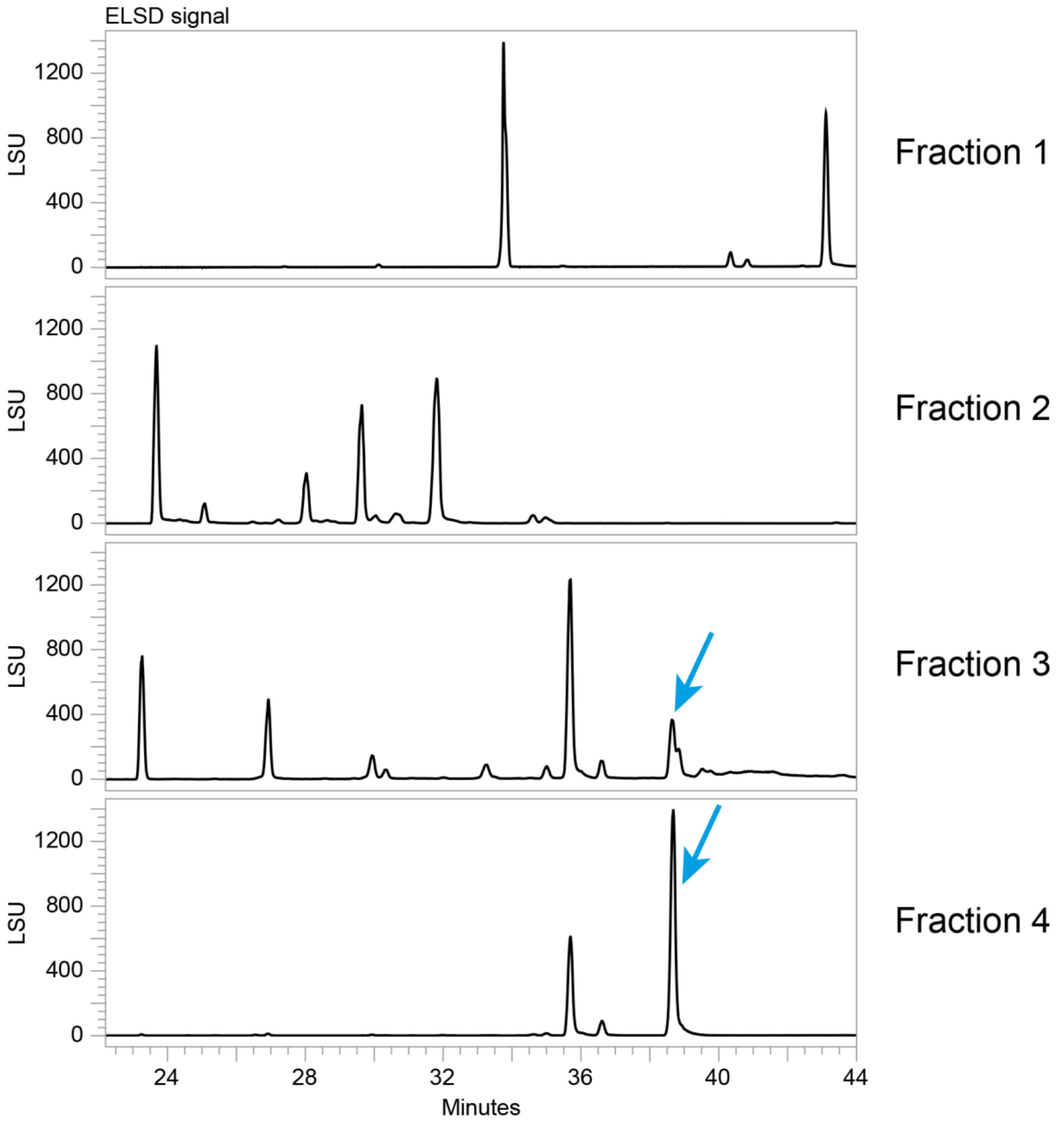

Supplementary Figure S2 – HPLC-ELSD comparison of four fractions of an active crude extract of *Colletotrichum higginsianum*. LSU, light scattering unit; ELSD, evaporative light scattering detector. Blue arrows = higginsianin B.

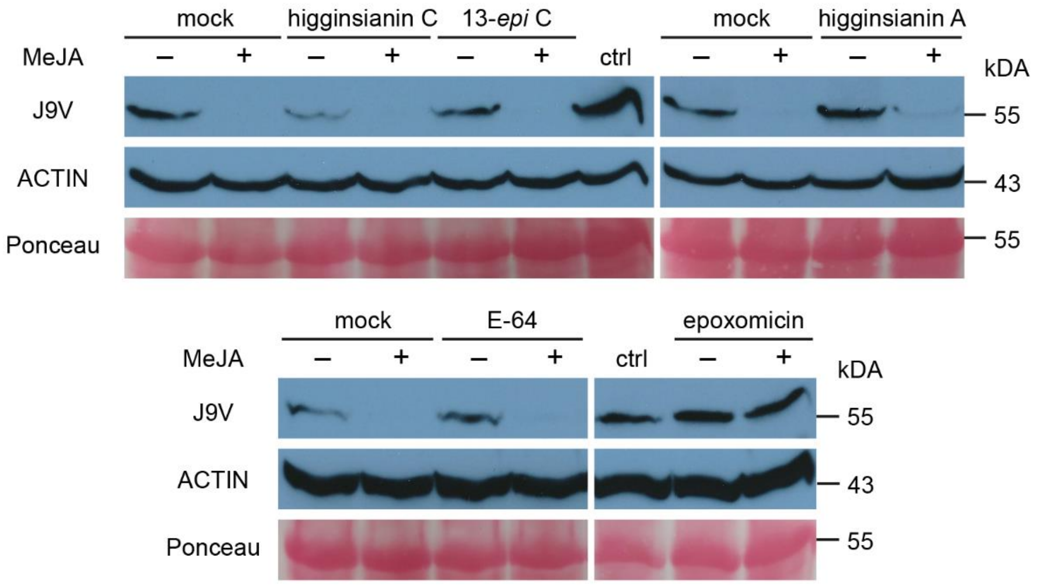

Supplementary Figure S3 – Pre-treatments with compounds structurally related to higginsianin B (higginsianins A, C and 13-*epi*-higginsianin C) do not influence the MeJA-induced degradation of the JA sensor J9V. Seedlings were pre-treated with either mock or 30 µM of the indicated compound for 30 min, following with a further 30 min treatment with either mock or 30 µM MeJA. Immunoblots depict 40 µg of total protein extracts from 60 seedlings. J9V was assayed with anti-GFP antibodies; ACTIN (assayed with anti-actin antibodies) and Ponceau S represent loading controls. Protein molecular mass is shown on the right. Higginsianins A, C and 13-*epi*-higginsianin C show very weak activity if any. ctrl refers to untreated Jas9-Venus seedlings. E-64 and epoxomicin, respectively a highly selective cysteine protease inhibitor and a specific proteasome inhibitor, were used as controls. Only epoxomicin could prevent MeJA-induced Jas9-VENUS degradation.
